# Computed tomography signs of intracranial hypertension: what intensivists should know

**DOI:** 10.62675/2965-2774.20260027

**Published:** 2026-07-24

**Authors:** Pedro Fortes Osório Bustamante, Filipe Matheus Cadamuro, Fabio Silvio Taccone, Pedro Kurtz, Thiago Gomes Romano

**Affiliations:** 1 Instituto D’Or de Pesquisa e Ensino Department of Intensive Care Medicine Rio de Janeiro RJ Brazil Department of Intensive Care Medicine, Instituto D’Or de Pesquisa e Ensino - Rio de Janeiro (RJ), Brazil.; 2 Universidade de São Paulo Intensive Care Unit Hospital das Clínicas São Paulo SP Brazil Intensive Care Unit, Hospital das Clínicas, Faculdade de Medicina, Universidade de São Paulo - São Paulo (SP), Brazil.; 3 Hôpital Universitaire de Bruxelles Université Libre de Bruxelles Department of Intensive Care Brussels Belgium Department of Intensive Care, Hôpital Universitaire de Bruxelles, Université Libre de Bruxelles - Brussels, Belgium.; 4 Neurointensive Care Unit Hospital São Luiz Itaim São Paulo SP Brazil Neurointensive Care Unit, Hospital São Luiz Itaim - São Paulo (SP), Brazil.

## INTRODUCTION

Non-contrast head computed tomography (CT), together with clinical examination, is the cornerstone of initial acute brain injury evaluation (i.e., traumatic brain injury, ischemic stroke, subarachnoid and intracerebral hemorrhage) due to its availability, rapid acquisition, and high sensitivity and specificity for detecting most acute intracranial pathologies.^(1-3)^

Nevertheless, timely access to radiologists for image interpretation remains limited, even in high-income countries.^(4,5)^ Even in centers with on-site radiology coverage, interpretation of CT-scan by intensive care unit (ICU) physicians may improve diagnostic accuracy and accelerate decision-making, particularly in patients at risk of elevated intracranial pressure (ICP). Trained non-radiology physicians may interpret CT scans with good accuracy.^(6)^

This viewpoint aims to guide intensivists in interpreting head CT, with emphasis on recognizing key imaging findings of intracranial hypertension and understanding its limitations.

### Increased intracranial pressure findings at computed tomography scan

Some CT findings are associated with intracranial hypertension. We highlight key imaging features and their performance in isolation; multiple concurrent findings increase the likelihood of elevated ICP. Importantly, interpretation should consider timing and clinical context, as these findings are not pathognomonic, and overtreatment of isolated findings may worsen outcomes.

### Basal cisterns effacement

Basal cisterns are cerebrospinal fluid (CSF)-filled spaces around the midbrain. In normal conditions, the CSF surrounding the midbrain facilitates clear identification of the midbrain and cerebral peduncles. In elevated ICP conditions, CSF is displaced toward extracranial compartments, impairing visualization of midbrain anatomy. This finding has a sensitivity of 86% and a specificity of 61% to detect elevated ICP.^(7)^

### Midline shift

Midline shift, evaluated on axial slices from CT-scan using the pineal body or septum pellucidum as reference structure, demonstrates displacement of cerebral structures and is correlated with elevated ICP. While limited (< 5mm) displacement is non-specific, moderate (5 - 10mm) and severe (> 10mm) shifts are associated with intracranial hypertension (sensitivity of 50% and 20%, and specificity of 70 and 90%, respectively).^(7)^

### Alterations in the lateral ventricles and enlargement of the temporal horn

The lateral ventricles may appear displaced by focal mass lesions or compressed and slit-like through mechanisms similar to basal cistern effacement.^(8)^ Although associated with elevated ICP, the diagnostic reliability of these findings remains uncertain.

Mass effect or ventricular hemorrhage may impair CSF circulation, leading to enlargement of the temporal horn of the lateral ventricle and hydrocephalus.^(9)^ Transependymal edema indicates increased intraventricular pressure. However, objective measures and prognostic value are not well established.

### Cerebral edema

Cerebral edema, either vasogenic or cytotoxic, results from multiple etiologies and leads to parenchymal swelling. In addition to basal cisterns effacement and ventricular compression, loss of grey-white matter differentiation and sulcal effacement are characteristic features associated with edema.^(10,11)^ The likelihood of elevated ICP increases with the number of concurrent imaging findings.^(7,11)^

### Herniation

Displacement of cerebral parenchyma may occur in several forms.^(12,13)^ Herniation syndromes of great relevance to intensivists include:

- "Subfalcine herniation" results from a midline shift. In severe cases, contralateral hydrocephalus due to obstruction of the foramen of Monro or infarction in the anterior cerebral artery territory may occur.
- "Central transtentorial herniation" and "lateral transtentorial herniation" (uncal herniation) occur when mass effect forces the uncus and adjacent structures (lateral) or the diencephalon (central) against the midbrain, potentially causing brainstem ischemia, Duret hemorrhage, and compression of the ipsilateral posterior cerebral artery.
- "Ascending transtentorial herniation" occurs when posterior cranial fossa lesions cause displacement of the cerebellum through the tentorial notch, resulting in compression of the posterior cerebral and superior cerebellar arteries and obstructive hydrocephalus.
- "Tonsillar herniation" occurs when the cerebellar tonsils descend below the foramen magnum, causing brainstem compression and often cardiorespiratory collapse.

### Systematic analysis of computed tomography scans by intensivists

**Figure 1:**
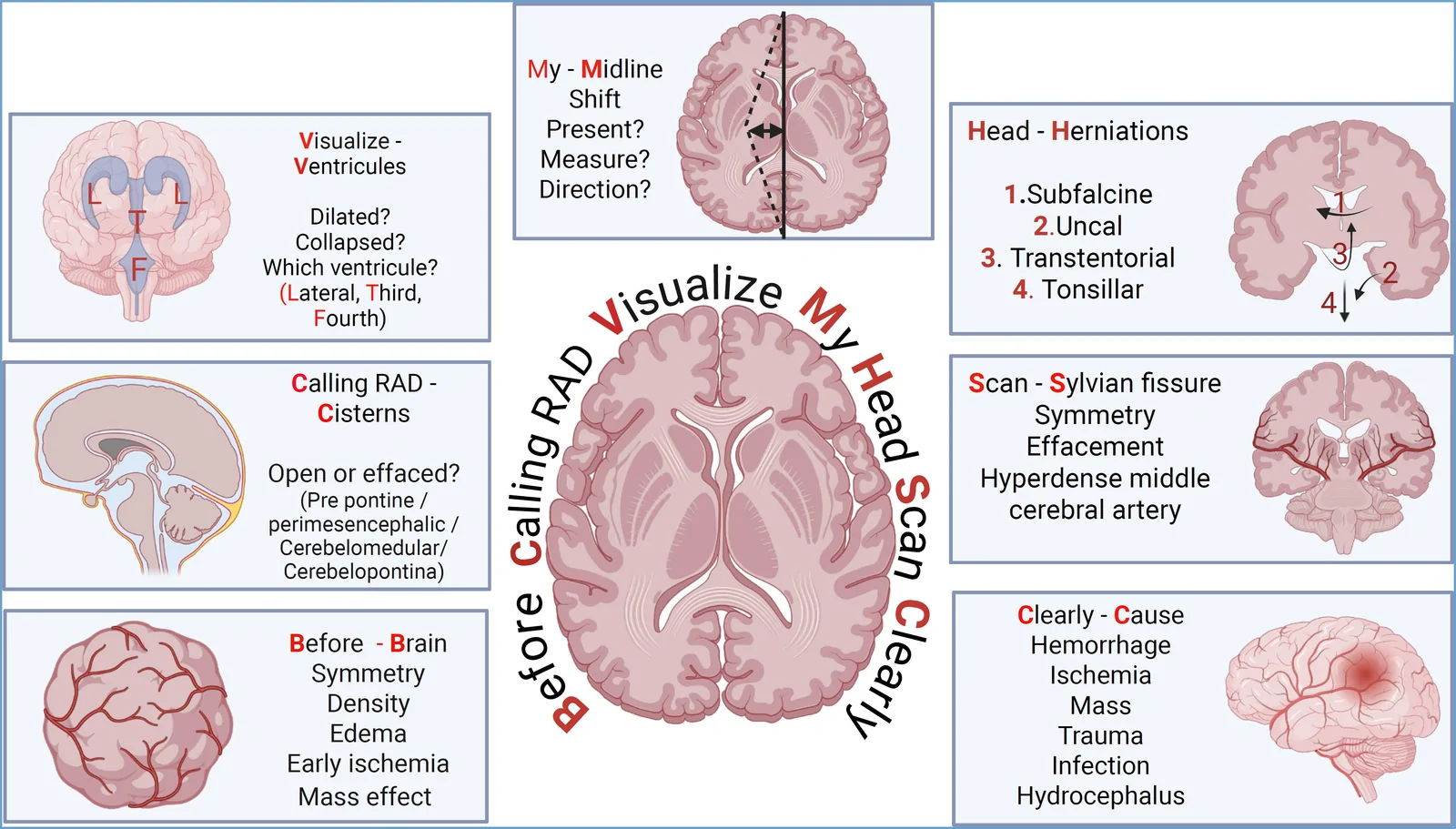
A systematic approach to computed tomography-scan analysis in the context of acute brain injury.

In acute brain injury, multiple lesions may increase the risk of missing subtle yet clinically relevant findings, even for experienced physicians.^(14)^ To mitigate this risk, we recommend a systematic approach to CT-scan analysis (Figure 1). Any structured review should include a deliberate search for the imaging findings described above. We propose the following simple mnemonic to reduce the risk of missing critical abnormalities:

Figure created by authors with Scientific Image and Illustration Software (BioRender, https://biorender.com).

**Before Calling the radiologist, Visualize My Head Scan Clearly**

**Before = Brain:** inspect the cerebral parenchyma for areas of hypo- or hyper-density. Hypodensities may indicate infarcts or edema, while hyperdensities may indicate hemorrhage (more rarely calcifications). In the absence of preexisting lesions, the brain parenchyma should appear symmetrical. Loss of clear grey–white matter differentiation may indicate diffuse cerebral edema.

**Calling = Cisterns:** evaluate the basal cisterns, particularly the peri-mesencephalic cisterns, which are normally well visualized. Effacement of these cisterns suggests elevated ICP and impairs clear identification of the midbrain surrounding structures.

**Visualize = Ventricles:** analyze the lateral ventricles (including the temporal horns), as well as the third and fourth ventricles. Both ventricular enlargement and effacement may be associated with elevated ICP. Hydrocephalus typically results in ventricular dilation, whereas diffuse cerebral edema or mass effect can compress the ventricles, except in the temporal horns, which may paradoxically dilate.

**My = Midline:** trace a line between the anterior and posterior attachments of the falx cerebri or along the superior sagittal sinus. The distance between this line and midline reference structures, most commonly the septum pellucidum, and alternatively the pineal gland, represents the degree of midline shift.

**Head = Herniation:** cerebral parenchyma may be displaced. The above-mentioned herniations are key indicators of elevated ICP and may cause secondary injury through vascular compression and mass effect. Careful evaluation of the different regions is essential to identify early signs of herniation.

**Scan = Sylvian fissure:** the Sylvian fissures should be clearly visible and symmetrical. Fissure obliteration may indicate cerebral edema. A hyperdense middle cerebral artery sign may be present in cases of acute arterial occlusion.

**Clearly = Cause:** the intensivist should seek the underlying cause of the abnormal findings. Hypodense regions following vascular territories suggest ischemic stroke, while vasogenic edema reflects blood–brain barrier disruption. Space-occupying lesions causing mass effect might warrant a surgical evaluation.

## Conclusions

Head computed tomography-scan remains a cornerstone in the management of acute brain injury, and intensivists should acquire proficiency in its systematic interpretation and recognize signs of intracranial hypertension during training to enable faster, more accurate clinical decision-making and improve patient care.

## Data Availability

Not applicable. This manuscript is a narrative viewpoint article and does not report original datasets, software code, or other research materials requiring public availability.
